# GDF15 promotes EMT and metastasis in colorectal cancer

**DOI:** 10.18632/oncotarget.6205

**Published:** 2015-10-22

**Authors:** Chen Li, Jingyu Wang, Jianlu Kong, Jinlong Tang, Yihua Wu, Enping Xu, Honghe Zhang, Maode Lai

**Affiliations:** ^1^ Department of Pathology, School of Medicine, Zhejiang University, Zhejiang, PR China; ^2^ Department of Pathology, the First Hospital of Jiaxing, Zhejiang, PR China; ^3^ Department of Pathology, the Second Affiliated Hospital, Zhejiang University School of Medicine, Zhejiang, PR China; ^4^ Key Laboratory of Disease Proteomics of Zhejiang Province, Zhejiang, PR China; ^5^ Key Constructing Discipline by Zhejiang Province and Jiaxing City, Zhejiang, PR China

**Keywords:** GDF15, EMT, metastasis, colorectal cancer

## Abstract

Metastasis is the major cause of cancer deaths, and the epithelial–mesenchymal transition (EMT) has been considered to be a fundamental event in cancer metastasis. However, the role of growth differentiation factor 15 (GDF15) in colorectal cancer (CRC) metastasis and EMT remains poorly understood. Here, we showed that GDF15 promoted CRC cell metastasis both *in vitro* and *in vivo*. In addition, the EMT process was enhanced by GDF15 through binding to TGF-β receptor to activate Smad2 and Smad3 pathways. Clinical data showed GDF15 level in tumor tissues, and the serum was significantly increased, in which high GDF15 level correlated with a reduced overall survival in CRC. Thus, GDF15 may promote colorectal cancer metastasis through activating EMT. Promisingly, GDF15 could be considered as a novel prognostic marker for CRC in the clinic.

## INTRODUCTION

GDF15 is a divergent member of the BMP- subfamily of the TGF-β superfamily, which is also designated as macrophage inhibitory cytokine-1 (MIC-1), prostate-derived factor (PDF), placental bone morphogenetic protein (PLAB), placental transforming growth factor (PTGF) and nonsteroidal anti-inflammatory drug-activated gene-1 (NAG-1) [1–5]. GDF15 is widely distributed in mammalian tissues and has multiple functions in various pathologies, including inflammation, cancer, cardiovascular diseases and obesity [6]. In endometrial [7], prostate [8], pancreatic [9], and colorectal [10] cancers, the circulating levels of GDF15 were elevated, which may correlate with poor clinical outcomes. However, the biological roles of GDF15 in tumorigenesis remain poorly understood and sometimes contradictory [11, 12]. GDF15 serves as a negative prognostic marker in CRC, and high level GDF15 both in tumor tissues and plasma correlate with an increased risk of recurrence and reduced overall survival [10, 13, 14]. Although GDF15 has been considered as the target for CRC therapy in some studies [15, 16], the biological function is not clear so far.

Our previous study identified GDF15 as the potential biomarker of lymph node metastasis in CRC using LC-MS/MS-based label-free quantitative proteomics approach [17]. Otherwise, GDF15 was also considered as a candidate biomarker for CRC diagnosis, which may be related to liver metastasis [18]. Nevertheless, the functional mechanism of GDF15 in CRC metastasis progression remains largely unknown. In this study, we investigated the functional mechanism of GDF15 in CRC metastasis. We clarified that GDF15 promoted EMT and metastasis in CRC through activating TGF-β pathway.

## RESULTS

### GDF15 promotes EMT and metastasis in colorectal cancer cell line

**Figure 1:**
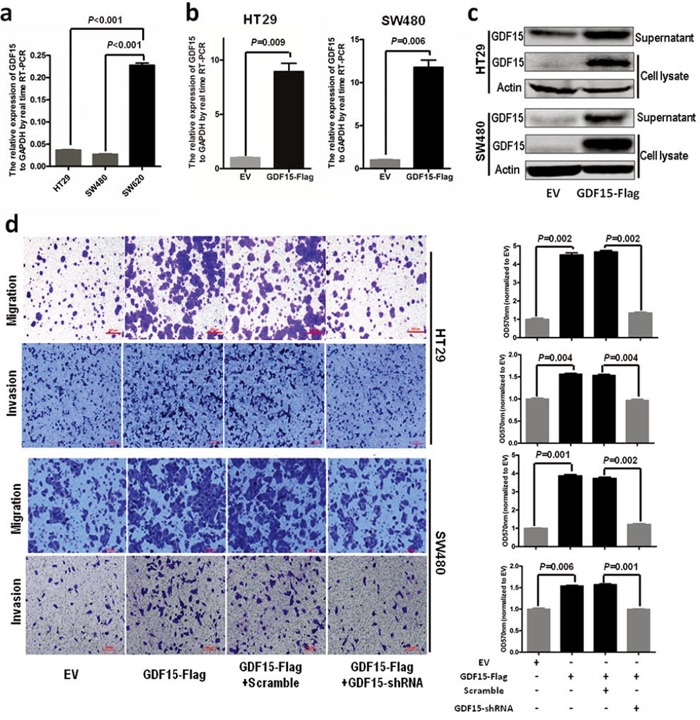
GDF15 promotes metastasis in HT29 and SW480 cells **a.** The expression of GDF15 in colorectal cancer cell lines. **b.** Detection of GDF15 in HT29 cells and SW480 cells with GDF15 overexpression by qRT-PCR. **c.** Detection of GDF15 in HT29 cells and SW480 cells with GDF15 overexpression by immunoblotting. **d.** Migration and invasion assay for HT29 and SW480 with GDF15 overexpression (quantification in right panels).

**Figure 2:**
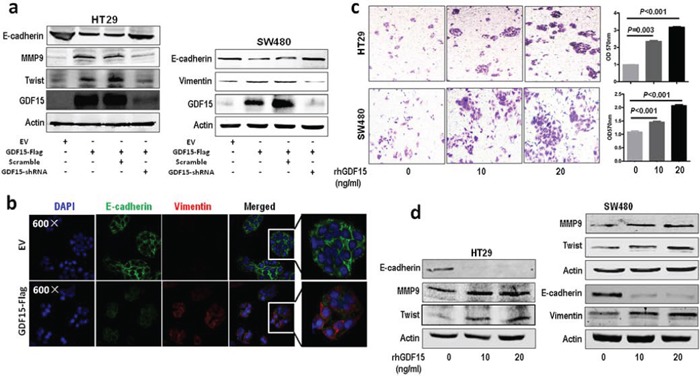
GDF15 promotes EMT in HT29 and SW480 cells **a.** Immunoblotting assay for expression of E-cadherin, MMP9, Twist and vimentin in GDF15 overexpression HT29 and SW480 cells. **b.** Immunofluorescence assay for expression of E-cadherin and vimentin in GDF15 overexpression SW480 cells. **c.** Migration assay for HT29 and SW480 cells with rhGDF15 treatment (quantification in right panels). **d.** Immunoblotting for E-cadherin, MMP9, Twist and vimentin in HT29 and SW480 cells with rhGDF15 treatment.

To investigate the molecular function of GDF15 in colorectal cancer, we detected the expression of GDF15 in colorectal cancer cell lines. RT-PCR showed that GDF15 expressed lower in HT29 and SW480 than in SW620 (Fig. 1a). So we transfected GDF15-Flag expressed vector to SW480 and HT29 cells (Fig. 1b and Fig. 1c) and determined whether ectopic expression of GDF15 could increase metastasis through EMT. Compared with empty vector (EV), GDF15-Flag vector promoted colorectal cancer cell migration and invasion (Fig. 1d). More interestingly, GDF15-Flag vector led to a significant reduction in E-cadherin as well as increases in the MMP9 and Twist expression levels in HT29 cells, and also significant increases in Vimentin in SW480 cells (Fig. 2a); Immunofluorescence assay also validated that GDF15 caused the changes in the expression of these EMT markers in SW480 cells (Fig. 2b). Since it is well known that GDF15 is a secreted protein, we used recombinant GDF15 protein (rhGDF15) to test whether GDF15 promoted EMT and metastasis through paracrine signal in colorectal cancer cells. As shown in Fig. 2c, rhGDF15 promoted cell migration in HT29 and SW480 cells. Moreover rhGDF15 repressed the expression of E-cadherin and enhanced the expression of vimentin, MMP9 and Twist (Fig. 2d). Taken together, these data demonstrated that GDF15 could promote EMT and metastasis by autocrine and paracrine signaling pathways in colorectal cancer.

### GDF15 knockdown in colorectal cancer cells inhibit EMT and metastasis

**Figure 3:**
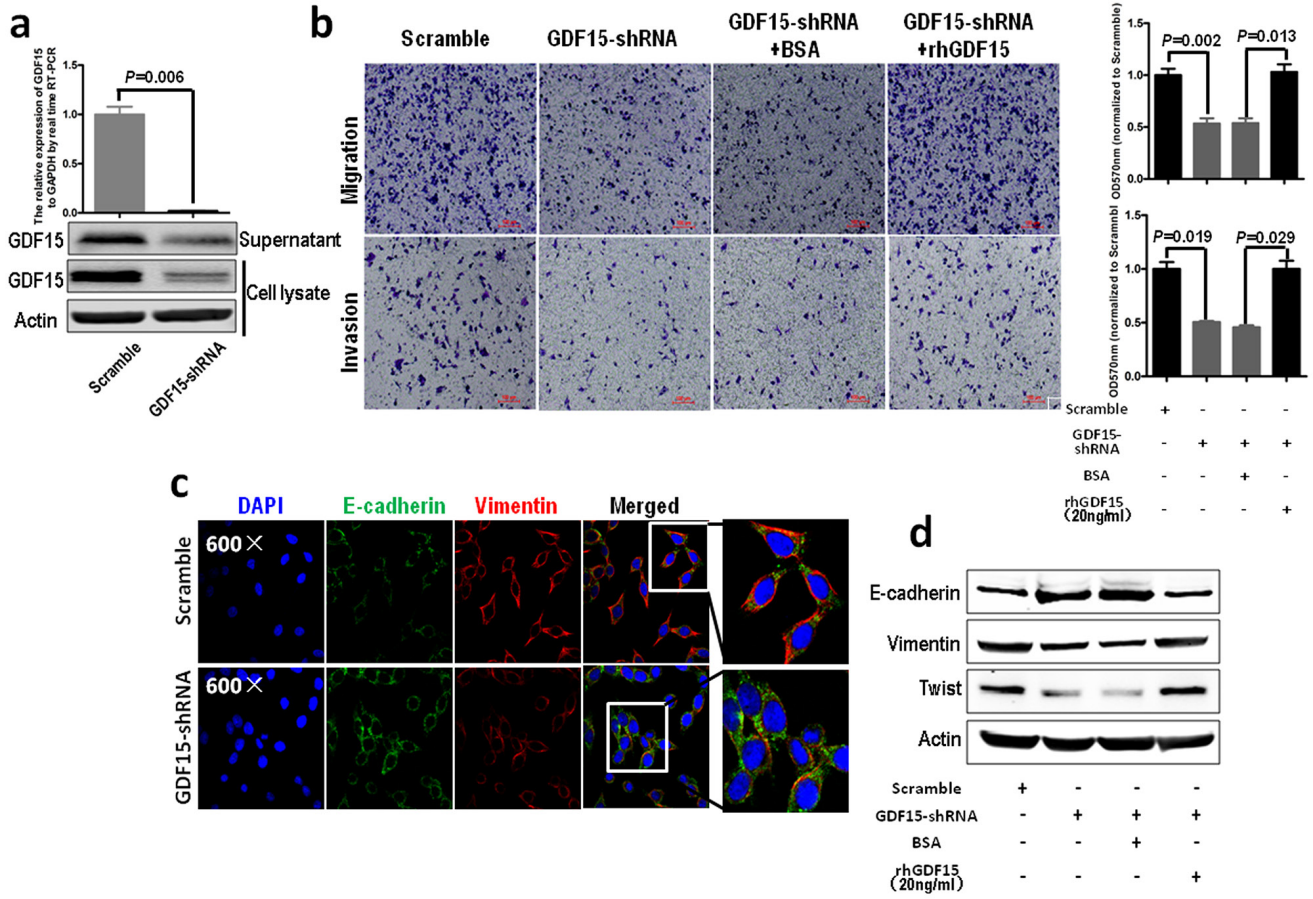
GDF15 knockdown in colorectal cancer cells inhibits EMT and metastasis **a.** Knockdown of GDF15 in SW620 cells using shRNA; upper panel represents RT-PCR and lower panel represents immunoblotting. **b.** Migration (upper panel) and invasion (lower panel) assay for SW620 with GDF15 shRNA (quantification in right panels). **c.** Immunofluorescence assay for SW620 with GDF15 shRNA. **d.** Immunoblotting assay for expression of E-cadherin, vimentin and Twist in GDF15 knockdown SW620 cells.

Next, we used lentivirus-mediated shRNAs to knock down GDF15 in SW620 cells, which showed that the expression of GDF15 was significantly reduced by GDF15-shRNA in both mRNA and protein levels (Fig. 3a). Meanwhile, GDF15-shRNA significantly represses migration and invasion of SW620 cells, and rhGDF15 could rescue the potential of migration and invasion of SW620 cells with GDF15-shRNA (Fig. 3b). GDF15 knockdown led to significant increases in E-cadherin as well as reduction in vimentin expression levels in SW620 cells (Fig. 3c and 3d). More interestingly, GDF15-shRNA not only could recover the alteration of migration and invasion caused by overexpression of GDF15 in SW480 and HT29 cells (Fig. 1d) but also could rescue the expression of E-cadherin, vimentin, MMP9 and Twist in SW480 and HT29 cells with GDF15-Flag (Fig. 2a). However, rhGDF15 rescued the expression changes of E-cadherin, vimentin and Twist, which were caused by GDF15 shRNA in SW620 cells (Fig. 3d). We concluded from these experiments that GDF15 promotes EMT and metastasis, at least in part, through regulating EMT.

### GDF15 promotes EMT through activating TGF-β signal pathway

**Figure 4:**
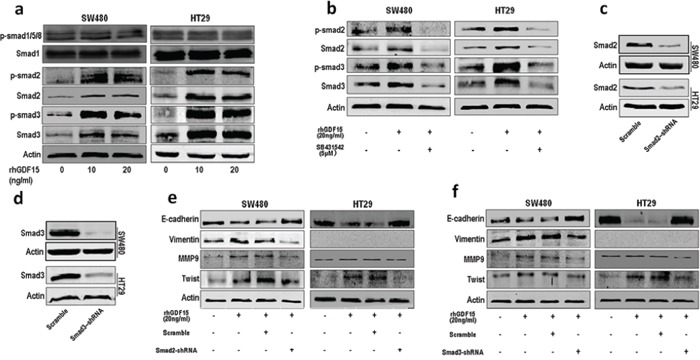
GDF15 regulates EMT through activating TGF-β signal pathway **a.** The expression and phosphorylation level of Smad1/5/8, Smad2 and Smad3 in SW480 and HT29 cells with rhGDF15 treatment. **b.** The expression and phosphorylation level of Smad2 and Smad3 in SW480 and HT29 cells with TGF-β receptor inhibitor SB431542 treatment. **c.** Knockdown of Smad2 in SW480 and HT29 cells. **d.** Knockdown of Smad3 in SW480 and HT29 cells. **e.** The rescue assay for E-cadherin, vimentin, MMP9 and Twist expression in SW480 and HT29 cells using Smad2 shRNA. **f.** The rescue assay for E-cadherin, vimentin, MMP9 and Twist expression in SW480 and HT29 cells using Smad3 shRNA.

GDF15 is a distant member of the bone morphogenetic protein (BMP) subfamily of the transforming growth factor-beta (TGF-β) superfamily. To confirm whether it could activate TGF-β signal pathway, we detected the expression of Smad2 and Smad3. The results showed that rhGDF15 promoted expression and phosphorylation of Smad2 and Smad3 in SW480 and HT29 cells (Fig. 4a). However, phosphorylation level of Smad1/5/8 was not changed by rhGDF15. Then we blocked the TGF-β receptor using SB431542, which antagonized the upregulated expression and phosphorylation of Smad2 and Smad3 caused by GDF15 in SW480 and HT29 cells (Fig. 4b). Furthermore, the shRNA was used to knock down Smad2 and Smad3 (Fig. 4c and 4d). Both Smad2 shRNA and Smad3 shRNA could rescue the expression of the epithelial marker E-cadherin and recover the expression of mesenchymal markers such as vimentin, MMP9 and Twist (Fig. 4e and 4f). Collectively, GDF15 promoted EMT through activating TGF-β signal pathway.

### GDF15 promoted metastasis *in vivo*

**Figure 5:**
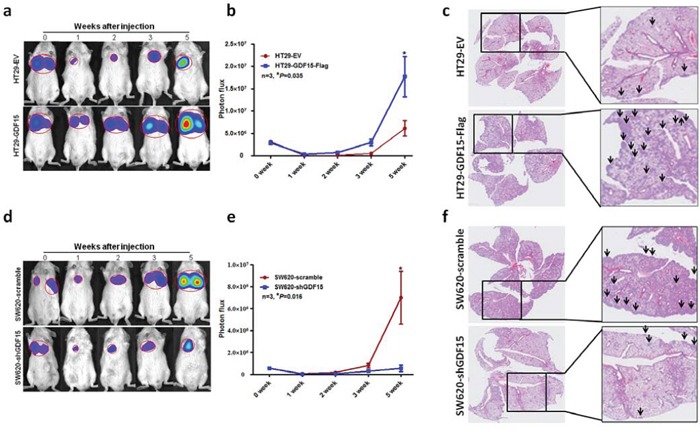
GDF15 promoted metastasis *in vivo* **a.** Representative images of luciferase signals and **b.** quantification of photon flux for metastases by tail-vein injection of HT29-EV and HT29 GDF15 overexpression cells in immunodeficient mice. **c.** HE staining for pulmonary metastatic foci from HT29-EV and HT29 GDF15 overexpression cells (The arrows indicated the metastatic foci). **d.** Representative images of luciferase signals and **e.** quantification of photon flux for metastases of SW620 scramble and SW620 GDF15 knockdown cells in immunodeficient mice. **f.** HE staining for pulmonary metastatic foci from SW620 scramble and SW620 GDF15 shRNA cells (The arrows indicated the metastatic foci).

To explore whether GDF15 could also increase metastasis *in vivo*, we intravenously injected luciferase-labelled control or GDF15-overexpressed HT29 cells into NOD/SCID mice and subjected them to bioluminescent imaging (BLI). 5 weeks after injection, the luminescence signals were about 4-fold higher for HT29 GDF15 overexpression cells than control cells (Fig. 5a), and continued BLI monitoring revealed a further increase in metastatic outgrowth in the lungs of mice injected with GDF15-overexpressing HT29 cells (Fig. 5b). HE staining showed that overexpression of GDF15 had largely promoted colonization of the lung with HT29 cells (Fig. 5c). To further confirm that GDF15 promotes metastasis *in vivo*, we injected GDF15 knockdown SW620 cells intravenously into NOD/SCID mice and monitored the signal within 5 weeks. GDF15 knockdown cells exhibited reduced lung metastasis abilities (Fig. 5d and 5e). The number of metastatic foci was also significantly decreased in the lung with injection of SW620 shGDF15 cells (Fig. 5f). Overall, data from these mice model strongly support the role of GDF15 as a pro-metastatic gene in colorectal cancer.

### Correlation between GDF15 and clinical characterization

**Figure 6:**
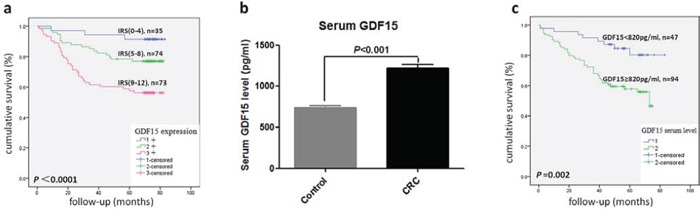
GDF15 level correlated with overall survival in CRC patients **a.** Kaplan–Meier plot of CRC specimens (*n* = 182) with different IRS scores of GDF15 by immunohistochemistry. A log-rank test indicated statistical significance (*P* < 0.0001). **b.** The level of serum GDF15 in healthy control and CRC patients (*P* < 0.001). **c.** Kaplan–Meier plot of CRC patients (*n* = 141) with high (≥820 pg/ml) and low (<820 pg/ml) serum levels of GDF15. A log-rank test indicated statistical significance (*P* = 0.002).

To test the hypothesis that GDF15 may be associated with an increased metastatic capacity of colorectal cancer, we detected the expression of GDF15 in a cohort of 234 primary colorectal cancer samples using immunohistochemistry (IHC). The elevated GDF15 expression showed a significant correlation with lymph node metastasis (*P* < 0.05, Table 1) and distant metastasis (*P* < 0.05, Table 1). In addition, a significant correlation between GDF15 expression and the tumor TNM grade was found (*P* < 0.01, Table 1). However, no correlation between age, gender and tumor size was detected. More interestingly, there was a significant correlation between GDF15 expression and tumor budding, which was considered as the cells with EMT phenotype in tumor tissues (*P* < 0.01, Table 1). Furthermore, we analyzed the survival data of these samples, and the results showed that tumors with the highest GDF15 expression were associated with the shortest overall patient survival, whereas patients with tumors displaying intermediate or low-grade GDF15 expression showed a better clinical outcome (*P* < 0.01, Table 2 and Fig. 6a).

**Table 1:** Relationship between GDF15 expression and clinicopathologic characteristic of CRC patients

Characteristic	Number*	Expression of GDF15			*P*
		Low	Moderate	High	
**Age (yr)**					0.428
≤60	102	15	46	41	
>60	132	28	57	47	
**Gender**					0.549
Male	126	20	58	48	
Female	108	23	45	40	
**Site**					0.399
Colon	119	20	46	53	
Rectum	96	19	43	34	
**Size (cm)**					0.428
≤5	123	19	50	54	
>5	86	18	37	31	
**Histological type**					0.325
Tubular adenocarcinoma	160	28	62	70	
Others	53	11	25	17	
**General type**					0.776
Ulcerated	132	23	52	57	
Others	70	15	27	28	
**Differentiation**					0.192
Well/moderate	177	33	83	61	
Poor/undifferentiated	57	10	20	27	
**Lymph node metastasis**					**0.014**
Negative	121	28	52	41	
Positive	90	9	36	45	
**Distant metastasis**					**0.024**
Negative	178	37	73	68	
Positive	27	0	12	15	
**TNM stage**					**0.002**
I/II	116	30	48	38	
III/IV	97	9	39	49	
**Tumor budding**					**0.008**
Negative	152	36	69	47	
Positive	67	6	28	33	

* Some data were missed.

**Table 2:** Univariate survival analysis of CRC

Variable	Numbers*	Cases of death	*P*
**Age (yr)**			0.122
≤60	88	20	
>60	107	20	
**Gender**			0.372
Male	110	29	
Female	85	26	
**Site**			0.073
Colon	107	35	
Rectum	86	18	
**Size (cm)**			0.621
≤5	111	29	
>5	76	23	
**Histological type**			0.910
Tubular adenocarcinoma	143	38	
Others	46	13	
**General type**			0.508
Ulcerated	111	33	
Others	72	18	
**Differentiation**			**0.001**
Well/moderate	146	34	
Poor/undifferentiated	46	21	
**Lymph node metastasis**			**<0.0001**
Negative	117	20	
Positive	73	33	
**Distant metastasis**			**<0.0001**
Negative	161	33	
Positive	22	17	
**TNM stage**			**<0.0001**
I/II	114	15	
III/IV	78	37	
**Tumor budding**			**0.033**
Negative	122	28	
Positive	51	19	
**Expression of GDF15**			**<0.0001**
IRS 1–4	35	3	
IRS 5–8	74	17	
IRS 9–12	73	32	

* Some data were missed.

GDF15 is a secretory protein, so we detected GDF15 level in plasma from another cohort of 287 colorectal cancer patients and 353 healthy control donors by ELISA. The CRC set showed significantly higher plasma levels of GDF15 compared with the healthy controls set (Fig. 6b). Then, we analyzed the association between plasma GDF15 level and clinical characterizations in CRC patients, which showed a significant correlation between high plasma GDF15 levels and differentiation, lymph node metastasis, distant metastasis and TNM grade (*P* < 0.01, Table 3). Multivariate Cox analysis revealed GDF15 as an independent prognostic factor (*P* = 0.039, Table 4). More importantly, the patients with high plasma GDF15 level displayed a poor prognosis (Table 3 and Fig. 6c).

**Table 3:** Relationship between serum GDF15 level and clinicopathologic characteristic of CRC patients

Characteristic	case* (%)	GDF15 level (pg/ml)		*P*
		Mean ± SD	Median	
**Age (yr)**				0.208
≤60	138(48.9)	1148.1 ± 651.0	992.3	
>60	144 (51.1)	1303.8 ± 886.1	1116.2	
**Gender**				0.081
Male	159 (55.4)	1266.0 ± 749.0	1148.9	
Female	128 (44.6)	1167.8 ± 812.6	989.1	
**Site**				0.416
Colon	142 (51.6)	1219.1 ± 718.1	1105.5	
Rectum	133 (48.4)	1243.3 ± 815.5	992.5	
**Size (cm)**				0.582
≤5	94 (72.9)	1167.0 ± 775.2	982.0	
>5	35 (27.1)	1324.1 ± 1006.5	995.2	
**Histological type**				0.631
Tubular adenocarcinoma	97 (87.4)	1246.7 ± 911.6	990.2	
Others	14 (12.6)	1172.7 ± 461.8	1078.8	
**General type**				0.934
Ulcerated	84 (72.4)	1137.2 ± 754.5	982.0	
Others	32 (27.6)	1299.5 ± 1049.8	970.7	
**Differentiation**				**<0.0001**
Well/moderate	212 (85.1)	1116.9 ± 597.0	989.1	
Poor/undifferentiated	37 (14.9)	2011.9 ± 1206.6	1409.47	
**Lymph node metastasis**				**<0.0001**
Negative	142 (52.6)	993.5 ± 517.5	916.0	
Positive	128 (47.4)	1527.5 ± 896.0	1409.4	
**Distant metastasis**				**0.005**
Negative	231 (81.9)	1170.9 ± 643.5	1049.0	
Positive	51 (18.1)	1604.7 ± 1124.8	1148.9	
**TNM stage**				**<0.0001**
I/II	127 (46.5)	973.8 ± 562.3	878.8	
III/IV	146 (53.5)	1491.2 ± 857.1	1354.1	
**Prognosis**				**0.001**
Survival	92 (64.8)	1043.2 ± 549.5	919.8	
Death	50 (35.2)	1557.0 ± 1210.6	1179.2	

* Some data were missed.

**Table 4:** Results of multivariate COX proportional hazard model

Variable	Relative risk (95% confidence interval)	*P*
Age	1.415 (0.764–2.621)	**0.269**
TNM stage	3.651 (1.819–7.331)	**<0.0001**
Expression of GDF15		**0.039**
IRS 1–4	Reference	
IRS 5–8	2.528 (0.563–11.342)	
IRS 9–12	4.740 (1.080–20.807)	

## DISCUSSION

Cancer metastasis is the major cause of human cancer-related death [19]. The intrinsic properties and microenvironments of tumor cell play the key role in dissemination and metastasis of cancer cells [20]. In this present study, GDF15-Flag vector increased migration and invasion in CRC cells, which demonstrated that GDF15 might promote CRC metastasis by an autocrine pattern. In addition, GDF15 might contribute to tumor progression through paracrine manners [21–23], because the exogenous rhGDF15 also could enhance the potential of metastasis in CRC. Our previous study showed a predominant expression of GDF15 in tumor nest not in stoma in CRC with a heterogeneous staining pattern. But the key source for paracrine GDF15 is still unclear, which should be remained to further study.

GDF15 could promote human prostate cancer cells metastasis through FAK-RhoA pathway [24], however we found that GDF15 could promote colorectal cacner metastasis through activating EMT process. More interestingly, GDF15 activated smad2 and smad3 through TGF-β I type receptor; moreover, smad2 or smad3 knockdown could rescue the EMT phenotype induced by GDF15. This study thereby demonstrates, for the first time, that GDF15 can promote EMT and metastasis through TGF-β receptor to activate smad2 and smad3 in colorectal cancer cells. Furthermore, we found that GDF15 level in tumor tissues and serum was significantly increased, in which high GDF15 level correlated with a reduced overall survival in CRC. Recently, GDF15 have been considered as a negative prognostic biomarker in colorectal cancer [13], gastric cancer [25], oral squamous cell carcinoma [26] and prostate cancer [27]. Nevertheless, GDF15 could also have antitumorigenic and proapoptotic activities, in which expression was found to be regulated by the PI3K/AKT/GSK-3β pathway and the tumor suppressors EGR-1and p53 [28–30]. Although *in vitro* assays show contradictory results, the fact that GDF15 shows dual functions in carcinogenesis is not surprising. As same as TGF-β, GDF15 may also act as a double-edge sword during the tumor development process. In the tumor initiation stage, it is able to suppress the proliferation of normal and premalignant epithelial cells. However, upon accumulation of genetic and epigenetic alterations in tumor cells, it switches to promotion of a proinvasive and prometastatic phenotype, accompanied by a progressive increase in the locally secreted GDF15 levels [31, 32]. Our data showed that high level of GDF15 in either tumor tissue or serum from CRC patients was positively correlated with metastasis and poor prognosis, which demonstrated that GDF15 might function as a prometastatic factor.

In conclusion, our findings reveal that a new pathway by GDF15 promotes EMT-mediated metastasis through activating TGF-β signaling cascade. Although larger clinical studies are needed to confirm these results, along with further research GDF15 may be considered as a novel prognostic marker for CRC in the clinic.

## MATERIALS AND METHODS

### Cell lines and cell culture

The human CRC cell lines SW480, SW620 and HT29 were purchased from the American Type Culture Collection (Manassas, VA, USA), which were maintained in RPMI 1640 medium; HEK293T cells were maintained in high-glucose DMEM. These cells were routinely maintained and supplemented with 10% fetal bovine serum (HyClone, Tauranga, New Zealand) and grown at 37°C in an atmosphere of 95% air and 5% CO_2_.

### DNA constructs and recombinant human GDF15 protein

All oligos, shRNA and primers are included in the supplementary materials. The full CDS sequence of GDF15 was amplified and cloned into p3xFLAG-CMV-14 (Sigma). The pLKO.1 lentivirus vector was used to construct shRNA vector, and lentiviruses were generated by co-transfecting 293T cells as previously described [33]. All transfection experiments were performed using Lipofectamine 2000 (Invitrogen), according to the manufacturer's instructions. In all, 10 ng/ml of recombinant human GDF15 protein (Peprotech, 120–28) was used to treat cells with 70%–80% confluence in RPMI 1640 medium without serum for 72 hours *in vitro*.

### RT-PCR

The total RNA was extracted from cells using TRIzol (Invitrogen), and cDNA was synthesized using PrimeScript^®^ RT regent kit (Takara Biotechnology, Dalian, China). Real-time PCR was performed with SYBR^®^ Premix Ex Taq (Takara Biotechnology, Dalian, China). The relative gene expression levels were calculated using the 2^−ΔCt^ method.

### Immunoblotting, immunohistochemistry and immunofluorescence

Immunoblotting, immunohistochemistry (IHC) and immunofluorescence (IF) studies were carried out as described previously [34]. The intensity of immunohistochemical reactions was appraised using the semi-quantitative immunoreactive score (IRS) scale of Remmele and Stegner. The information of all antibodies was followed: GDF15 (Abcam; 1:1000 for WB, 1:400 for IHC), E-cadherin (Santa Cruz Biotechnology Inc.; 1:200 for WB, 1:500 for IF), Vimentin (Cell Signaling Technology; 1:1000 for WB, 1:500 for IF), MMP9 (Cell Signaling Technology; 1:1000 for WB), Twist (Santa Cruz Biotechnology Inc., 1:200 for WB), Smad2/3, Smad1, p-Smad2/3, p-Smad1/5/8 (Cell Signaling Technology, 1:1000 for WB) and β-actin (Santa Cruz Biotechnology Inc., 1:5000 for WB). β-actin was used as a loading control for western blots.

### Cell migration and invasion assay

To evaluate the migration and invasion capacity of cells, 24-well plates equipped with cell culture inserts containing 8.0 μm pore size membrane (Costar Corp., Cambridge, MA, USA) were used. Briefly, cells were suspended in 100 μl of serum-free medium and placed in the upper compartment chambers. The lower chamber was filled with 10% FBS as the chemoattractant. For invasion assay, diluted extracellular matrix gel (BD Biosciences, Bedford, MA, USA) coated the inserts preincubated for 30 min. In all, 5 × 10^4^ cells incubated for the migration assay and 1 × 10^5^ cells incubated for the invasion assay. At the end of the experiments, cells on the upper surface of the filters were wiped, and migrated cells on the lower surface were fixed in 4% paraformaldehyde and stained by 0.1% crystal violet.

### Clinical data

Two independent cohorts of patients were enrolled with informed consent under institutional review board-approved protocols of Zhejiang University. In all, 234 paraffin-embedded tissue samples from sporadic colorectal adenocarcinoma were obtained from one group of patients without any adjuvant treatment including radiotherapy or chemotherapy prior to surgery and diagnosis. Another group of CRC patients provided 287 serum samples in the Sir Run Run Shaw Hospital, Zhejiang University. A total of 353 healthy control subjects were obtained from routine healthy examinations during the same period. All patients received no preoperative chemotherapy or radiotherapy. The serum samples were liquated into RNase-free tubes after centrifugation at 4°C. The serum was kept at −80°C and thawed before analyses.

### ELISA assay

The concentrations of GDF15 in serum were detected by using sandwich enzyme-linked immunosorbent assays (ELISA, catalog number MAB957, R&D Systems, Inc., Minneapolis, MN, USA) according to the manufacturer's instructions.

### Animal experiments

5–6 weeks severe combined immunodeficient mice (NOD–SCID–gamma) were used for animal studies. Animal experiments were approved by the Animal Ethics Committee of Zhejiang University, China. Control cells and GDF15 overexpression or knockdown cells with pLenti PGK V5-LUC Neo vector (1 × 10^6^ cells) suspended in 0.1 ml PBS were intravenously injected into the tail vein. Post intraperitoneal injections of 1.5 mg of luciferin (Gold Biotechnology) for 10 min, the metastases were monitored using the IVIS@ Lumina II system (Caliper Life Sciences, Hopkinton, MA, USA). On account of excessive tumor burdens, all animals were humanely sacrificed after 5 weeks. Pieces of lung were fixed in 10% formalin before embedded in paraffin. Serial sections of the embedded specimens were stained with hematoxylin and eosin (H&E) as conventionally conducted.

### Statistical analysis

Each experiment was performed in triplicate and repeated at least three times. Data were presented as mean ± standard deviation (SD). The statistical analyses were conducted with SPSS 20.0. *P*-values <0.05 were considered statistically significant.

## SUPPLEMENTARY TABLE


