# Supplementary material for: GDF15 promotes EMT and metastasis in colorectal cancer
Source: Oncotarget. 2015 Oct 22;7(1):860–72. doi: 10.18632/oncotarget.6205 (PMC4808038; doi:10.18632/oncotarget.6205)
Supplement: Supplementary file 1 [file oncotarget-07-0860-s001.pdf]

SUPPLEMENTARY TABLE

Supplementary Table S1: Oligas used in this study

Oligos	Sequence
GAPDH-primer-F	ACCACAGTCCATGCCATCAC
GAPDH-primer-R	TCCACCACCCTGTTGCTGTA
GDF15-primer-F	TCAGATGCTCCTGGTGTTGC
GDF15-primer-R	GATCCCAGCCGCACTTCTG
GDF15-shRNA-F	CCGGGCTCCAGACCTATGATGACTTCTCGAGAAGTCATCATAGGTCTGGAGCTTTTTG
GDF15-shRNA-R (TRCN0000058388)	AATTCAAAAAGCTCCAGACCTATGATGACTTCTCGAGAAGTCATCATAGGTCTGGAGC
SMAD2-shRNA-F	CCGGCGATTAGATGAGCTTGAGAAACTCGAGTTTCTCAAGCTCATCTAATCGTTTTTG
SMAD2-shRNA-R (TRCN0000040036)	AATTCAAAAACGATTAGATGAGCTTGAGAAACTCGAGTTTCTCAAGCTCATCTAATCG
SMAD3-shRNA-F	CCGGCATCTCCTACTACGAGCTGAACTCGAGTTCAGCTCGTAGTAGGAGATGTTTTTG
SMAD3-shRNA-R (TRCN0000020012)	AATTCAAAAACATCTCCTACTACGAGCTGAACTCGAGTTCAGCTCGTAGTAGGAGATG
